# Is Obesity More Than a Double Burden among People with Mobility Disability? The Effect of Obesity on HRQoL and Participation in Society

**DOI:** 10.3390/healthcare5040079

**Published:** 2017-10-24

**Authors:** Marianne Holmgren, Jeroen de Munter, Finn Rasmussen, Magnus Sandberg, Gerd Ahlström

**Affiliations:** 1 Department of Health Sciences, Lund University, P.O. Box 157, Lund SE-221 00, Sweden; finn.rasmussen@med.lu.se (F.R.); magnus.sandberg@med.lu.se (M.S.); gerd.ahlstrom@med.lu.se (G.A.); 2 Statistics Sweden, P.O. Box 24300, Stockholm SE-104 51, Sweden; Jeroen.DeMunter@scb.se

**Keywords:** mobility disability, disabled person, overweight, obesity, health-related quality of life, participation in society, public health

## Abstract

Obesity is more common in individuals with mobility disability than in those without this condition. Individuals with mobility disability also have lower health-related quality of life (HRQoL) and are limited in their participation in society. Therefore, this study aimed to investigate the body mass index (BMI) status and the association of overweight or obesity on HRQoL and participation in society among those with mobility disability in comparison to those without mobility disability. This cross-sectional study was based on a health survey conducted in Sweden in 2012 (*n* = 18,322; age, 18–64 years). Logistic regression with and without interaction analysis was applied. Effect modification by overweight status was significant for, moderate pain. For obesity, effect modification was seen for low general health, pain (moderate and severe), and not participating in work. BMI was higher among those with mobility disability, but no associations between overweight or obesity and HRQoL or participation in society were observed for those with mobility disability. Overweight and obesity did not add an additional burden to mobility disability, probably because mobility disability is associated with low HRQoL and low participation in society. Despite these results, population obesity prevention strategies are still needed.

## 1. Introduction

About 15% of the global adult population aged 15 years and over is estimated to live with some kind of disability, and between 2.2–3.8% have significant difficulties in functioning [1]. In Sweden, the corresponding prevalence for those aged 16–84 years with disability is 21%, and 7% have difficulties in functioning [2]. According to the International Classification of Functioning, Disability and Health (ICF), disability is an umbrella term for impairments, activity limitations, and participation restrictions, reflecting a complex interaction between a person’s impairments of bodily functions and the characteristics of their environment [3]. Difficulties in functioning are related to lower health-related quality of life (HRQoL) [4,5], which is defined as an individual or group’s perception of physical and mental health [6]. Moreover, participation or an individual’s involvement in daily life is important for people with disabilities and in the field of disability research in order to allow for example health planning [3]. The United Nations has also highlighted the importance of participation, as they have stated it to be a human right [7]. Obesity is also related to lower HRQoL [8], and the prevalence of obesity has more than doubled globally in the last four decades [9]. Obesity is even more common among individuals with disability [10,11,12]. It is now well-documented that overweight and obesity are associated with increased morbidity and mortality [13,14]. Thus, both participation and obesity are important factors that could influence HRQoL in people with disabilities. 

Individuals with mobility disability (MD) are a vulnerable group with poor health status compared to those without MD, and with increased prevalence of comorbidities including obesity [15,16]. There is a gap of knowledge about how obesity influences HRQoL in those with MD, and if there is more than a “double burden” among those in which both conditions are present. Since both obesity and MD separately are linked to low HRQoL, an additional risk of low HRQoL might be expected when MD and overweight or obesity coexist. Measuring HRQoL in a population gives an insight into the nation’s health [6], and therefore, knowledge that explores the “double burden” for people with mobility disability will be important in the guidance of performing health promotion. Moreover, the prevalence of obesity among individuals with MD is sparsely explored in Sweden, and therefore more studies are needed on this topic. Such new knowledge might enable the evaluation of whether current prevention efforts are sufficient to reduce the amount of people with obesity and guide further work on health promotion strategies for people with MD.

In the same way as MD affects HRQoL, it is well-known that individuals with MD are limited in their participation in society [3]. However, obesity as a predictor of participation in society is not well researched. Previous research shows that obesity has negative associations with participation in society, for example working life [17] and also daily living including leisure time [18]. However, the burden of obesity on participation in society among individuals with MD has not been well explored. The aim of this study was to investigate the BMI status and the association of overweight or obesity on HRQoL and participation in society among those with MD in comparison to those without MD.

## 2. Materials and Methods

### 2.1. Design and Context

This study was a cross-sectional population study based on data from the Skåne Public Health Survey (SPHS) in Sweden, collected by Statistics Sweden between October 2012 and March 2013. The SPHS data was used to identify study populations and to study outcomes. 

### 2.2. Study Population

**Figure 1:**
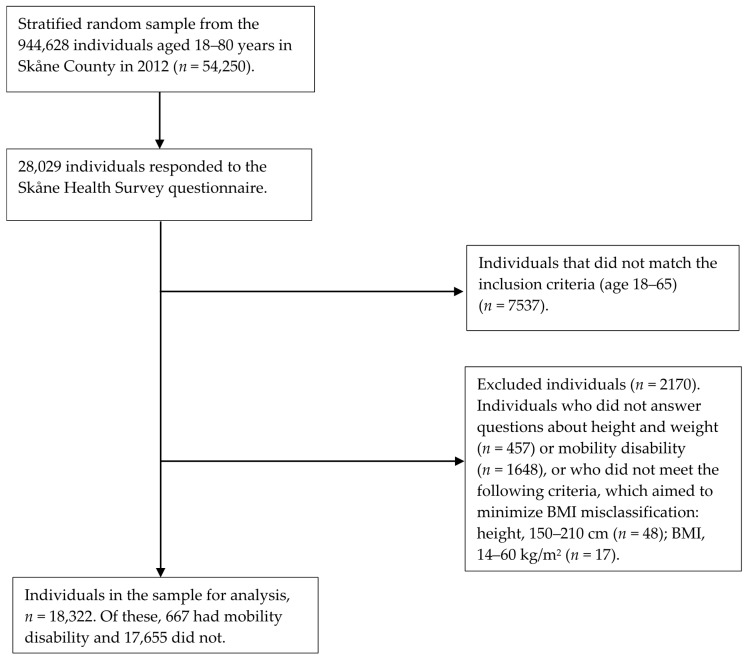
Flowchart for participating individuals in the Skåne Public Health Survey 2012. BMI: body mass index.

Out of 944,628 individuals aged 18–80 in Skåne County in 2012, a stratified random sample (*n* = 54,250) was selected for the public health survey (see Figure 1). The response rate was 51.7%, or 28,029 individuals. The population between 18 and 64 years included 18,322 individuals, of whom 667 had MD. 

Three questions were used to identify individuals with MD. Those that stated that they were unable to run 100 m in combination with either taking one single step on a stair and/or taking a short walk lasting approximately 5 min. The Public Health Agency of Sweden [12] has used this definition in previous surveys. Questions about weight and height were used to define BMI (kg/m^2^). We defined underweight (≤18.5 kg/m^2^), normal weight (18.5 to 25 kg/m^2^), overweight (25.0 to 30 kg/m^2^), and obesity (≥30 kg/m^2^) in line with the World Health Organization (WHO) classification [16]. Underweight and normal weight were merged because of the data scarcity (18 of 321 or 5.5% had MD).

### 2.3. Measurements

Outcome variables related to HRQoL: HRQoL was assessed by five self-rated dimensions: general health and vitality (from SF-36), mental health (GHQ12), pain (EQ5D), and sleep problems. The question on general health [17] asked “How would you rate your general health?”, with five possible answer alternatives: “Excellent”, “Very good”, “Good”, “Poor”, and “Very poor”. Answers of “Poor” or “Very poor” were considered to be poor general health. Vitality was measured using four items with six possible answer alternatives, scoring 4–24 points. Transformation of raw scale scores gave a range from 0–100 [17], and we categorized individuals in “low vitality” or “high vitality” using the mean value for the Swedish general population, with 68.8 as a cut-off. The widely-used 12-item General Health Questionnaire (GHQ12) instrument [18] was used to measure mental health, with a cut-off score of more than 2 and a maximum of 12 considered poor mental health. Participants marked their current level of pain based on one item in the EQ5D instrument [19]. The three alternatives were “I have no pain or discomfort”, “I have moderate pain or discomfort”, and “I have extreme pain or discomfort”. Sleep problems were assessed by two questions, the first one was “Do you think you get enough sleep to feel rested?” (with possible answers of “Yes, often”, “Yes, but not enough”, and “No, never or almost never”) and the second question was “During the past 14 days, have you had trouble with insomnia or sleeping badly and if so, to what extent?” (with the possible answers of “Yes, quite often”, “Yes, a little”, and “No”). The responses “No, never or almost never” for the first question and the “Yes, quite often” to the second were considered to define a sleep problem.

Outcome variables related to participation in society: Participation in society included measurements of their social participation and their participation in work. Social participation was measured through a 13-item instrument that measures how actively a person takes part in activities of formal and informal groups in society [19]. Respondents were asked to answer if they, during the last 12 months had: participated in a study circle/course at work; participated in a study circle/course in their spare time; participated in a union meeting; participated in meeting of an association; visited the theatre/cinema; visited an art exhibition; participated in a religious session; been to a public sports event; written a letter to a newspaper/magazine; taken part in a demonstration of some sort; visited a place of public entertainment (e.g., night club, dance, etc.); taken part in a large family gathering or been to a party at someone’s home. We considered a cut-off score of ≤3 activities as low social participation. Participation in work was measured by one question about current employment. The alternatives were: in work, approved absence/parental leave, studying/in training, measure for entering labor market, unemployed, retired, on disability pension, on long-term sick leave (more than three months), or other. Individuals who answered that they were working, but formally absent from work or on parental leave, and/or studying were defined as individuals participating in work. Others were defined as not participating in work.

### 2.4. Statistics

An independent samples *t*-test was conducted to compare the BMI scores for individuals with and without MD. Analyses were performed to compare those with and without MD and to assess the effect of BMI within each group, using normal weight/underweight as the reference category. For each outcome, odds ratios (ORs) with 95% confidence intervals (CIs) were estimated using logistic regression. Potential effect modification was investigated using an interaction term in the logistic regression model. All analyses were adjusted for the background variables gender, age, education level, marital status, and country of birth because of the known differences in the literature between individuals with and without MD.

Dichotomous variables were created for MD (0 = no; 1 = yes), general health (0 = good; 1 = poor), pain using two dummy variables with no pain as reference (no pain vs. moderate pain, and no pain vs. extreme pain), mental health (0 = good; 1 = poor), vitality (0 = high; 1 = low), sleep problems (0 = no problems; 1 = problems), social participation (0 = high; 1 = low), and participation in work (0 = work; 1 = not work). Statistical analyses were performed using SPSS Version 22.0 (IBM Corp., Armonk, NY, USA) for Windows.

## 3. Results

Individuals with MD were more likely to be female, older, and obese (see Table 1) compared to those without MD. Individuals with MD also had lower levels of education, were less often in work, and more likely to have been born abroad, as well as more likely to be unmarried. Individuals with MD had a mean BMI of 28.3 kg/m^2^ (SD = 6.0) compared with individuals without MD, who had a mean BMI of 25.5 kg/m^2^ (SD = 4.3), *p* < 0.001 (not in table).

No statistically significant effects of overweight were found among people with MD (Table 2). In the group of people without MD, overweight carried an increased risk of pain (moderate as well as severe), low vitality, sleep problem and low social participation. Moreover, interaction analyses revealed that the effect of overweight on moderate pain was larger among those without than those with MD (Table 2).

No statistically significant effects of obesity were found among people with MD (Table 2). Obesity was associated with higher odds for all investigated outcomes among those without MD. Interaction analyses suggested that for low general health, pain (moderate and severe), and not participating in work, the ORs were consistently and statistically significantly higher in the group without MD compared to the group with MD (Table 2).

## 4. Discussion

To our knowledge, only one study has investigated the interaction between weight status and MD status on HRQoL and participation in society. That study was based on a population from a different part of Sweden, and our research team was unable to show any statistically significant interaction effects [20]. There remains a clear gap of knowledge in this specific area, and therefore the present study was necessary to broaden the evidence base, investigate the interaction in a more rural area, and to include participation. Since both conditions (obesity and MD) are separately associated with low HRQoL [4,8,20], we expected to see an additional risk for low HRQoL in those who have both conditions. However, being overweight or obese did not further decrease the HRQoL in individuals with MD. One possible reason is that individuals with MD already had low HRQoL and that overweight or obesity as additional factors did not further worsen HRQoL [4,5]. Furthermore, chronic pain, fatigue, and sleep problems are common secondary conditions among people with MD, and are also known to cause low HRQoL [15]. This means that these factors should also be taken into account in addition to weight status and the MD itself when trying to improve such a complex area as HRQoL in people with MD. Another reason for the absence of an association may be explained by the fact that the proportion of adults with class III obesity (above 40 kg/m^2^) and MD was low in our study (only 26 individuals), making it difficult to detect possible inverse associations between BMI and HRQoL in this group. Perhaps a higher proportion of individuals with class III obesity would have shown a statistically significant association. Most recent studies on MD and obesity have been carried out in the United States, where the rate of obesity is higher (about 35% [5,21]) compared to Sweden (around 14% [22]) and the other Nordic countries (10–16% [23]). Due to the different context, this study will contribute to a wider understanding of obesity among those with mobility disability in countries with similar rates of obesity, and is therefore of importance in obesity prevention.

The differences in characteristics among different weight classes have also been shown in other studies. A meta-analysis by Ul-Haq et al. showed significant differences between those with class III obesity and those with overweight or class I and II obesity (from 30 to 40 kg/m^2^) in terms of HRQoL and physical health [8]. Furthermore, Salem et al. [24] examined associations between obesity and HRQoL among individuals with disability (i.e., muscular dystrophy, multiple sclerosis, post-polio syndrome, and spinal cord injury) and the general population. Both obese and non-obese individuals with disability reported worse HRQoL in comparison to the general population sample, but within the disability groups, obese individuals with muscular dystrophy and multiple sclerosis reported worse HRQoL in several domains when compared to non-obese individuals [24]. Nevertheless, the differences between the obese and non-obese groups in terms of HRQoL were relatively small and may not be clinically relevant. This means that the association between weight class and HRQoL may depend on the obesity class. In addition, this might also depend on the type of disability diagnosis. Further research should therefore examine how different diagnoses are related to MD and different levels of obesity (class I, II, and III) and HRQoL. Despite the absence of an association with HRQoL in this study (intended to reveal a “double burden” of having both MD and obesity), people with MD may still experience this as a “double burden”. Qualitative research could be one way to increase our knowledge about the meaning of obesity to individuals with MD by exploring their own experience. The present study reveals that regardless of the absence of association to HRQoL in those with MD, they have still higher BMI than people without MD, and must therefore have continued attention to prevent obesity to avoid co-morbidity with serious obesity-related illnesses, but also attention to increase their HRQoL.

Participation in society was not associated with obesity or overweight among those with MD. Thus, the explanation for the absence of such effect may also be explained—as for HRQoL—by an already low participation in society for people with MD, such that obesity did not further worsen their participation in society. This new sample may imply that obesity in people with MD is not associated with low participation in society, which is an important contribution to this research area. However, there is no consensus on the definitions of participation in society, which can also be understood as structural social capital [25]. Social capital can also include family and friends, which is not the case in the present study where this result has to be interpreted with caution [26]. 

The SPHS survey received more responses from women, older individuals, and individuals with higher levels of education than in the general population [27]. In our sample, comparisons between those included (*n* = 20,492) and those excluded (*n* = 2170), Figure 1, showed no significant difference in gender and weight, but significant differences in age and education level. Those excluded were younger and had lower levels of education. The higher proportion of individuals with MD in the older group (those aged 57–64) is in line with national statistics, and one reason may be that disability tends to increase with age [28]. Women also tend to have higher levels of disability than men. However, the higher levels of obesity and lower HRQoL in individuals with MD and the higher levels of low HRQoL except for mental health in obese individuals without MD is in line with the previous literature [8,20,29], and it is therefore reasonable to believe that external dropout is not a great threat to external validity. 

There was no information available about the type of MD or whether MD or obesity had occurred first, which may have influenced the result. It might be that the association to HRQoL is different for those with MD who then became obese, in relation to those who were obese and then acquired MD. However, this cannot be investigated in the present study because of the cross-sectional study design, which is a limitation. The presence of the bidirectional association between body weight and MD has been studied by de Munter and colleagues, using a population-based sample from Stockholm, Sweden [30]. The study showed that people with MD at baseline increased more in BMI at the eight-year follow-up compared to people without MD, and also that people with overweight or obesity had a greater risk of reporting MD over the eight year period. However, we are not aware of any studies that have investigated these two subgroups (MD that became obese, and obese that acquired MD), to determine if there are any differences between them when it comes to HRQoL. Thus, we do not know how this affects the results in the present study, and the interpretations should therefore be made with this in mind. Future public health surveys should therefore contain specific questions about the origin of both MD and obesity. HRQoL draws on measurements of physical functions, such as walking and climbing, and its use has been criticized in individuals with MD who automatically receive a lower score for these questions [31,32]. We attempted to select measurements of HRQoL that were as function-neutral as possible, looking at general health, mental health, pain, vitality, and sleep problems. This gives greater opportunities to investigate HRQoL independently of physical functions, which is a strength of this study. The differences in methods used to measure HRQoL in individuals with MD could make it difficult to compare studies. This study, which has similarities with another study from the capital city of Sweden by the authors [20], contributes to the picture of obesity´s association to HRQoL in individuals with MD. However, the present study included both rural and urban areas, which makes the results more generalizable. In the previous study by the authors, the outcome variable for participation in society included both participation in activity and participation in work, but in the present study, social participation and participation in work are separated to make it possible to investigate potential differences.

One limitation of this study is that all data were assessed by self-reported measures, which could be a threat to internal validity. For example, self-reported data on weight and height are known to be less precise than the measured data [33], and therefore the prevalence of overweight status could be underestimated. It is also recognized that BMI may not be the best measure to use with individuals with MD because of their lower muscle mass [10]. Furthermore, the question about the mobility limitation did not reveal for how long the participants have had their mobility limitations, and therefore, some may only have had short-term limitations. This needs to be addressed in future studies.

## 5. Conclusions

Our findings indicate that obesity does not add an additional burden to MD, probably because MD already are associated to low HRQoL and low participation in society. Obesity is more common among individuals with MD compared to those without MD. Thus, both obesity prevention strategies and health promotion are necessary to prevent morbidity and promote healthy weight in the MD population. The level and/or type of MD and the level of obesity might play a significant role in both HRQoL and participation in society, and needs to be addressed in future research.

## Figures and Tables

**Table 1:** Population characteristics *n* = 18,322.

	All (*n* = 18,322)		MD (*n* = 667)		NMD (*n* = 17,655)	
	%	*n*	%	*n*	%	*n*
Sex, Female (%)	55.2	(10,119)	61.9	(413)	55.0	(9706)
Age, % (*n*)						
18–36 years	31.5	(5775)	8.2	(55)	32.4	(5720)
37–56 years	46.2	(8459)	44.2	(295)	46.2	(8164)
57–64 years	22.3	(4088)	47.5	(317)	21.4	(3771)
Marital status (%)						
Married	48.7	(8928)	50.5	(337)	48.7	(8591)
Unmarried	38.6	(7070)	22.5	(150)	39.2	(6920)
Divorced	11.6	(2020)	25.2	(168)	11.1	(1952)
Widow/Widower	1.1	(204)	1.8	(12)	1.1	(192)
Body mass index, mean (SD)	25.7	(4.32)	28.3	(6.0)	25.5	(4.3)
Underweight	1.8	(321)	2.7	(18)	1.7	(303)
Normal weight (%)	9.1	(8996)	29.2	(195)	49.8	(8801)
Overweight (%)	35.0	(6418)	34.2	(228)	35.1	(6190)
Obesity (%)	14.1	(2587)	33.9	(226)	13.4	(2361)
Education, % (*n*)						
Primary education (up to 9 years education)	13.3	(2440)	25.3	(162)	12.9	(2183)
Secondary education (10–12 years education)	42.0	(7699)	51.3	(329)	42.3	(7159)
Post-secondary education (more than 13 years education)	43.4	(7957)	23.4	(150)	44.9	(7601)
Employment status, % (*n*) ^1^						
In work	62.1	(11,376)	26.7	(178)	63.4	(11,198)
Parental leave	3.2	(593)	0.9	(6)	3.3	(587)
Study, practice	11.0	(2007)	3.1	(21)	11.2	(1986)
Measure for entering Labor markets	0.8	(153)	1.2	(8)	0.8	(145)
Unemployed	6.4	(1174)	10.0	(67)	6.3	(1107)
Retired	2.5	(450)	6.1	(41)	2.3	(409)
Disability pension	5.2	(946)	31.6	(211)	4.2	735
Long-term sick leave (more than 3 months)	1.9	(349)	14.5	(97)	1.4	(252)
Other	8.3	(1514)	15.4	(103)	8.0	(1411)
Country of birth, % (*n*)						
Sweden	83.6	(15,313)	68.7	(458)	84.1	(14,855)

MD: mobility disability; NMD: no mobility disability; ^1^ = More than one answer was allowed.

**Table 2:** Interaction effect between mobility disability and weight status and the association between mobility disability and health-related quality of life (HRQoL) and participations in society in different weight groups; *n* = 18,322.

	Mobility Disability			No Mobility Disability			*p*-Value for Interaction Overweight	*p*-Value for Interaction Obesity
	NW	OW	OB	NW	OW	OB		
	OR (CI)	OR (CI)	OR (CI)	OR (CI)	OR (CI)	OR (CI)		
**HRQoL**								
Low general health	1	1.07 (0.72,1.60)	0.96 (0.64,1.43)	1	1.19 (1.0,1.43)	2.62 **(2.17,3.17)**	0.602	**<0.001**
Low mental health	1	1.28 (0.84,1.96)	1.26 (0.83,1.92)	1	0.96 (0.88,1.05)	1.19 **(1.06,1.34)**	0.066	0.388
Pain, moderate	1	0.77 (0.52,1.15)	0.97 (0.65,1.45)	1	1.24 **(1.15,1.33)**	1.79 **(1.63,1.97)**	**0.006**	**0.001**
Pain, extreme	1	1.08 (0.71,1.64)	0.99 (0.65,1.50)	1	1.28 **(1.03,1.59**)	2.05 **(1.60,2.61)**	0.534	**0.002**
Low vitality	1	0.79 (0.41,1.51)	1.20 (0.56,2.23)	1	1.14 **(1.06,1.22)**	1.70 **(1.55,1.88)**	0.495	0.467
Sleep problem	1	0.90 (0.60,1.36)	0.93 (0.61,1.40)	1	1.13 **(1.03,1.24)**	1.47 **(1.30,1.65)**	0.403	0.054
**Participation in society**								
Low social participation	1	0.75 (0.48,1.18)	0.89 (0.56,1.40)	1	1.16 **(1.08,1.25)**	1.43 **(1.29,1.58)**	0,070	0.052
Not participating in work	1	1.06 (0.69,1.62)	0.90 (0.59,1.38)	1	1.06 (1.97,1.15)	1.33 **(1.20,1.48)**	0.852	**0.040**

NW: normal weight; OW: overweight; OB: obesity; OR: odds ratio; CI: confidence interval. Significant values are shown in bold text.
